# Strength and Power Performance Changes During an In-Season Resistance Training Program in Elite Futsal Players: A Case Study

**DOI:** 10.2478/hukin-2022-0096

**Published:** 2022-11-08

**Authors:** Diogo Luís Marques, João Nuno Ribeiro, António Carlos Sousa, Bruno Travassos, Mário Cardoso Marques

**Affiliations:** 1 Department of Sport Sciences, University of Beira Interior, Covilhã, Portugal.; 2 Research Center in Sports Sciences, Health Sciences and Human Development, CIDESD, Covilhã, Portugal.; 3 Portugal Football School, Portuguese Football Federation, Oeiras, Portugal.

**Keywords:** low volume, low-to-moderate loads, volume load, TQR, RPE

## Abstract

In this study, we aimed to analyze (i) the strength and power changes after resistance training (RT) in elite futsal players, and (ii) the associations between the session rate of perceived exertion (sRPE) and perceived total quality recovery (TQR), and the sRPE and TQR with the volume load of the RT program. Ten elite futsal players (24.8 ± 5.4 years; 76.2 ± 7.1 kg; 1.77 ± 0.05 m) performed an in-season 8-week RT program twice per week. RT consisted of 2-3 sets x 3-6 reps at 45-65% of one-repetition maximum (1RM) with maximal velocities in the full squat and complementary exercises with the same volume. We assessed the TQR before every session, while the sRPE was calculated after each RT session. One week before and after the intervention, we measured the countermovement jump (CMJ) height, isometric hip adduction strength (IHAS), 1RM, and peak power (PP) in the full squat progressive loading test. After the 8-week training program, there was a significant improvement in most outcomes, yet the gains (%Δ) remained below the minimal detectable change (MDC), except for IHAS (CMJ: p < 0.05, %Δ = 6.7, MDC% = 7.2; IHAS: p < 0.001, %Δ = 19.1, MDC% = 14.6; 1RM: p > 0.05, %Δ = 9.2, MDC% = 21.5; PP: p < 0.05; %Δ = 14.4, MDC% = 22.4). We also found a significant negative correlation between TQR and the sRPE (r = -0.45, p < 0.001). Our data suggest that RT based on low-volume and low-to-moderate loads may not produce a sufficient stimulus to induce meaningful dynamic strength and power gains in elite futsal players, although it improves isometric strength. Furthermore, monitoring TQR before sessions may show coaches how the elite futsal player will perceive the session's intensity.

## Introduction

Futsal is an intermittent high-intensity indoor team sport that requires players with high athletic performance to cope with match demands (Dogramaci et al., 2011; Ribeiro et al., 2020; Sekulic et al., 2021; Travassos et al., 2011). Recent exploratory data with elite futsal players show that the distance covered per minute, the number of sprints, decelerations, and metabolic power are the variables that best discriminate between the physical profiles of high-level players (Ribeiro et al., 2020). These physical abilities are crucial in executing specific futsal skills (e.g., kicking, dribbling, passing, ball recovery), and consequently, they are critical indicators of overall performance in futsal matches (Ribeiro et al., 2020; Sekulic et al., 2021). Therefore, strength and conditioning futsal coaches should guarantee that futsal players are integrated into complementary training programs to optimize high-intensity actions.

Previous studies have demonstrated the positive effects of resistance training (RT) programs on futsal players' physical performance (Marques et al., 2019; Paz-Franco et al., 2017; Radzimiński and Jastrzębski 2021; Torres-Torrelo et al., 2017). For example, Torres-Torrelo et al. (2017) observed that performing 2–3 sets x 4–6 reps at 45–60% of the one-repetition maximum (1RM) with maximal velocities in the full squat significantly improved strength, jump, sprint, and repeated sprint ability in futsal players competing in the Spanish third division. Similarly, Paz-Franco et al. (2017) observed that performing 3 sets x 8 reps at 75% 1RM with maximal velocities in the half squat, leg press, and hamstring curl significantly improved jump, sprint, and repeated sprint ability in professional futsal players. Nevertheless, in the latter, the authors did not consider the effects of the RT program on measures of muscle strength (e.g., 1RM), nor specified the division where players competed, making it challenging to infer about the transfer effect for players competing at the highest level (e.g., first division). Therefore, it is essential to conduct studies with elite futsal players competing at the highest level to make hypothetical inferences about the effects of RT on muscle strength and provide guidelines to optimize the design of RT in futsal, as it happens in other sports. For example, previous research with elite soccer players suggested short-term RT with progressively increasing loads over the intervention combined with complementary exercises (e.g., core exercises) to improve 1RM strength and vertical jump ability (Fischerova et al., 2021).

In addition to implementing an RT program and analyzing its effects on athletes' physical performance, monitoring both the internal and external workloads during the intervention enables understanding the player's response to the stimuli imposed (Helms et al., 2020; Hiscock et al., 2015, 2018). According to the literature, the session rate of perceived exertion (sRPE) appears to be a simple and effective strategy to monitor the internal training load of RT sessions (Helms et al., 2020; Hiscock et al., 2015, 2018). In this line, previous research with physically active men (at least one year of RT background) observed significant correlations between the sRPE and measures of external training load, such as the volume load (sets x reps x weight lifted) (Genner and Weston, 2014; Hiscock et al., 2018; Lodo et al., 2012). According to these observations, it is expected that the higher the volume load, the higher the sRPE would be, and vice-versa (Genner and Weston, 2014; Hiscock et al., 2018; Lodo et al., 2012). Nevertheless, to the best of our knowledge, there is no evidence regarding the associations between the sRPE and the volume load in elite futsal players, which emphasizes the need for further research on this topic. In addition, since training monitoring should also focus on player's readiness and recovery (Brink et al., 2010), understanding the associations between measures of perceived recovery and markers of internal and external training load (i.e., sRPE and volume load, respectively) should also be emphasized in future research, in particular with elite futsal players.

Therefore, given the considerations above, we aimed to (i) analyze the strength and power performance changes after an in-season 8-week RT program in elite futsal players, and (ii) examine the associations between the sRPE and perceived recovery, as well as between the sRPE and perceived recovery with the volume load of the RT program. Our hypotheses were that (i) the RT program would significantly improve strength and power performance during the in-season in elite futsal players, and (ii) the sRPE would be significantly associated with perceived recovery, as well as the sRPE and perceived recovery with the volume load of the RT program.

## Methods

### Study design

We conducted this case study with elite futsal players competing in the first division of the Portuguese Futsal League. We implemented the in-season 8-week RT program from January to March during the players’ gym sessions (Tuesday and Thursday between 10 and 11 a.m.). Strength and power performance was assessed before and after the 8-week intervention using the following tests: 1) a countermovement jump (CMJ) test, 2) an adductor squeeze strength test, and 3) a full squat progressive loading test. Two weeks before the pretest, all players were familiarized with the testing procedures and exercises. In addition, players performed the full squat progressive loading test one week before the pretest to assess the test-retest reliability. All players performed the tests in a fitness health club under the same environmental conditions (20–22°C) on Tuesday morning (10 to 12 a.m.). Players were instructed to maintain their habitual daily routines (i.e., hydration and nutrition) during the RT program.

### Participants

Ten elite futsal players participated in this study (age: 24.8 ± 5.4 years; body mass: 76.2 ± 7.1 kg; body height: 1.77 ± 0.05 m; futsal experience: 12.4 ± 3.9 years). All players regularly participated in 67 weekly futsal training sessions (1.22 ± 0.14 h per session), two weekly gym sessions (2 h per week), and one official match per week. In addition to the Portuguese Futsal League, players competed in two national knock-out competitions: the Portuguese Futsal Cup and the Portuguese Futsal League Cup. Although players had participated in gym sessions since the pre-season, none was engaged in a systematic RT program nor consistently performed RT for at least one year. Before starting the study, we fully informed futsal coaches, medical staff, and players about the possible benefits and risks of the RT program. The Ethical Committee of the University approved this study, and all players signed a written informed consent form.

### Procedures

All players performed a 20 min warm-up consisting of 15 min running at a self-selected pace on a treadmill or pedaling on a stationary bicycle, followed by 5 min of joint mobilization, 5 min of bodyweight squats (2 sets x 5 reps) and CMJs (2 sets x 3 reps). Two team coaches and one strength and conditioning coach supervised the warm-up and tests.

*Countermovement jump height test.* Players started the test upright, with their hands akimbo and the feet shoulder-width apart. After instruction, they flexed the knees at 90°, followed by a maximal vertical jump as high as possible, always maintaining their hands on the hips. All players performed three repetitions, interspersed by 30 s rest intervals (Marques et al., 2019). An infrared timing system (Optojump, Microgate, Bolzano, Italy) was used to estimate the jump height, and the maximum value reached (cm) was selected for further analysis. The intraclass correlation coefficient (ICC) was 0.94 (95% confidence interval (CI), 0.84–0.98), and the coefficient of variation (CV) was 2.6%.

*Adductor squeeze strength test.* Players lay down on a mat in a supine position with 45º hip flexion and 90º knee flexion. The dynamometer (Smart Groin Trainer, NeuroExcellence, Braga, Portugal) was placed between the player's knees and then attached to the thighs with Velcro straps (Moreno-Pérez et al., 2019; Sousa et al., 2022). The Smart Groin Trainer device has been shown to be a valid and reliable tool for measuring isometric hip adduction strength (IHAS) (Moreno-Pérez et al., 2019; Sousa et al., 2022). Before the test, players performed a specific warm-up of 3 submaximal isometric hip adduction contractions lasting 5 s, interspersed by 30 s rest intervals. After recovery, they performed three maximal isometric hip adduction contractions lasting 5 s, followed by 3 min rest intervals between subsequent attempts (Moreno-Pérez et al., 2019; Sousa et al., 2022). We used the maximum IHAS value (kg) for further analysis. The ICC was 0.95 (95% CI, 0.86–0.99), while the CV was 5.3%.

*Full squat progressive loading test.* All players performed the full squat on a Smith machine (G3-PL62, MATRIX Fitness, USA). They assumed an initial stance position with the barbell positioned on the trapezius, the knees and hips fully extended, and the feet at shoulder-width apart. When ready, they descended the barbell at a controlled velocity (2–3 s) until 35–45° knee flexion, and after a 1 s pause, they extended their knees at the maximal intended velocity. We allowed players to lift their heels at the end of the concentric phase, but not jump off the ground (Sánchez-Medina et al., 2017). We recorded each repetition's mean propulsive velocity (MPV) through a linear velocity transducer (T-Force System, Ergotech, Murcia, Spain). The specific warm-up consisted of 6 reps with 17 kg. All players started the test with 27 kg and the load was gradually increased by 10 kg until players attained an MPV of ~0.80 m·s^-1^, which corresponds to ~75% 1RM (Sánchez-Medina et al., 2017). Players performed three repetitions with each load and rested for 3 min between sets (Sousa et al., 2020). To estimate the 1RM (kg), we selected the fastest repetition attained against the last load and used the following equation: (100 × last load) / ((-2.185 x MPV^2^) + (-61.53 x MPV) + 122.5) (Torres-Torrelo et al., 2017). Additionally, the maximum peak power value (W) attained during the test was registered for further analyses. The ICC for the progressive loading test was 0.85 (95% CI, 0.50–0.96), while the CV was 7.7%.

*Resistance training program.* Players performed an RT protocol twice per week on nonconsecutive days (48 h rest) during the eight weeks, each session lasting 60 min. After a general briefing of the session led by the team's coach, players performed a 20 min warm-up consisting of 10 min running at a self-selected pace on a treadmill or pedaling on a stationary bicycle, 5 min of joint mobilization, and 5 min of bodyweight squats (2 sets x 5 reps) and CMJs (2 sets x 3 reps). Then, they performed the main part of the session (40 min). The principal exercise was the Smith Machine full squat. We prescribed low-volume (2–3 sets and 3–6 reps), low-to-moderate relative intensities (45–65% 1RM), and fast concentric actions (< 1 s) due to the reported benefits of this approach in futsal players (Marques et al., 2019; Torres-Torrelo et al., 2017). The inter-set rest interval was 2–3 min. We calculated each player's volume load in every session by multiplying the total repetitions by the weight lifted. Then, we summed each player's volume load to determine the total session volume load (Bird et al., 2013). Table 1 presents the structure of the full squat RT program. Players also performed complementary exercises, including the CMJ, Nordic hamstring curl, Copenhagen adductor, glute bridge, and core exercises (i.e., TRX plank to pike, dead bug, and frontal and side plank) (Table 2). The inter-set rest interval was between 30–60 s in the complementary exercises. One team coach and one strength and conditioning coach supervised all sessions.

**Table 1:** Full squat training program.

	Week 1		Week 2		Week 3		Week 4	
Full Squat	Session 1	Session 2	Session 3	Session 4	Session 5	Session 6	Session 7	Session 8
Sets x Reps	2 x 6	3 x 6	3 x 6	2 x 5	3 x 5	3 x 5	2 x 5	3 x 5
Intensity 1RM) (%	45	45	45	50	50	50	55	55
Weight (kg) Volume load (kg·reps)	42.3 ± 8.4 5074.4	42.3 ± 8.4 7611.7	42.3 ± 8.4 7611.7	47.0 ± 9.4 4222.6	47.0 ± 9.4 7047.8	47.0 ± 9.4 7047.8	51.7 ± 10.3 5168.4	51.7 ± 10.3 7752.6

	Week 5		Week 6		Week 7		Week 8	
Full Squat	Session 9	Session 10	Session 11	Session 12	Session 13	Session 14	Session 15	Session 16
Sets x Reps	3 x 5	2 x 4	3 x 4	3 x 4	2 x 3	3 x 3	3 x 3	2 x 4
Intensity (%1RM)	55	60	60	60	65	65	65	60
Weight (kg) Volume load (kg·reps)	51.7 ± 10.3 6417.3	56.4 ± 11.2 4510.6	56.4 ± 11.2 5446.0	56.4 ± 11.2 6077.0	61.1 ± 12.2 3291.7	61.1 ± 12.2 4937.6	61.1 ± 12.2 4385.2	56.4 ± 11.2 4051.3

RM: repetition maximum.

**Table 2:** *Complementary exercises in the training program*.

Exercises	Week 1 Session 1	Session 2	Week 2 Session 3	Session 4	Week 3 Session 5	Session 6	Week 4 Session 7	Session 8
CMJ (S x R)	2 x 6	3 x 6	3 x 6	2 x 5	3 x 5	3 x 5	2 x 5	3 x 5
Nordic (S x R)	2 x 6	3 x 6	3 x 6	2 x 5	3 x 5	3 x 5	2 x 5	3 x 5
Copenhagen (S x R)	2 x 6	3 x 6	3 x 6	2 x 5	3 x 5	3 x 5	2 x 5	3 x 5
Glute Bridge (S x R)	2 x 6	3 x 6	3 x 6	2 x 5	3 x 5	3 x 5	2 x 5	3 x 5
Core R) exercises (S x	2 x 6	3 x 6	3 x 6	2 x 5	3 x 5	3 x 5	2 x 5	3 x 5

	Week 5		Week 6		Week 7		Week 8	
Exercises	Session 9	Session 10	Session 11	Session 12	Session 13	Session 14	Session 15	Session 16
CMJ (S x R)	3 x 5	2 x 4	3 x 4	3 x 4	2 x 3	3 x 3	3 x 3	2 x 4
Nordic (S x R)	3 x 5	2 x 4	3 x 4	3 x 4	2 x 3	3 x 3	3 x 3	2 x 4
Copenhagen (S x R)	3 x 5	2 x 4	3 x 4	3 x 4	2 x 3	3 x 3	3 x 3	2 x 4
Glute Bridge (S x R)	3 x 5	2 x 4	3 x 4	3 x 4	2 x 3	3 x 3	3 x 3	2 x 4
Core exercises (S x R)	3 x 5	2 x 4	3 x 4	3 x 4	2 x 3	3 x 3	3 x 3	2 x 4

S: sets; R: repetitions; CMJ: countermovement jump; In the Nordic exercise, the time under tension was ~6 s; In the Copenhagen exercise, players performed the sets on each side, and the time under tension was ~3 s; In the frontal and side plank, players performed the same number of sets for 1 min.

*Total quality recovery and session rate of perceived exertion logs.* Approximately 10 min before each session, each player individually rated his perceived recovery using the 15-point total quality recovery (TQR) scale, which ranges from 6 (very, very poor recovery) to 20 (very, very good recovery) (Brink et al., 2010). All players were familiarized with the scale since the pre-season. Approximately 15 min after each RT session (Hiscock et al., 2015), each player individually rated the sRPE using the 15-point RPE scale, which ranges from 6 (very, very light) to 20 (very, very hard) (Brink et al., 2010). As for the TQR scale, all players were familiarized with the RPE scale since the pre-season.

### Statistical Analysis

Before conducting statistical tests, we checked the assumptions of normality using the Shapiro-Wilk test. Since all variables followed a normal distribution, parametric tests were applied. We used a paired samples *t*-test to analyze the pretest to posttest differences in all dependent variables. The magnitude of the effects was determined using the Hedge's *g* effect size and interpreted based on the recommendations for untrained individuals in RT: <0.50, trivial; 0.50–1.24, small; 1.25–2.00, moderate; >2.00, large (Rhea, 2004). The percent change from pretest to posttest was calculated with 90% CIs in all dependent variables (Δ% = ([Posttest – Pretest] / Pretest) x 100). In addition, Pearson correlation coefficients with 95% CI were calculated to determine the relationships between the sRPE with TQR and the sRPE and TQR with the volume load. The magnitude of correlations was interpreted as: 0.00–0.10, negligible; 0.10–0.39, weak; 0.40–0.69, moderate; 0.70–0.89, strong; 0.90– 1.00, very strong (Schober et al., 2018). The ICC_(2,1)_ analyzed the relative reliability (Koo and Li, 2016), while the standard error of measurement (SEM = standard deviation of the pretest x √1 - ICC_(2,1)_) and the CV ((SEM / mean of pretest) x 100) analyzed the absolute reliability (Lexell and Downham, 2005; Sainani, 2017). Reliability was considered acceptable when ICC values were ≥ 0.75 and CV values were ≤ 10% (Cormack et al., 2008; Koo and Li, 2016). To estimate the sensitivity to change, we calculated the minimal detectable change (MDC = √2 x SEM x 1.96) (Sainani, 2017) and MDC% ((MDC / mean of pretest) x 100) (Lexell and Downham, 2005). The alpha level was set at *p* < 0.05. All statistical analyses were conducted using Microsoft Office Excel (Microsoft Inc., Redmond, WA, USA) and SPSS v27 (SPSS Inc., Chicago, IL, USA). For plotting the data, we used the GraphPad Prism v7 (GraphPad Inc., San Diego, CA, USA).

## Results

### Resistance training program and training log

The overall attendance rate in the RT program was 100%. However, in the full squat exercise, the rate was 93% because when a player reported higher muscle fatigue than usual due to matches or futsal sessions, the staff consensually decided to exclude this exercise in the RT session. Over the RT program, the average volume load in the full squat was 5665.9 ± 1436.6 kg·reps, the average TQR was 15.1 ± 1.7 (i.e., good recovery), and the average sRPE was 12.4 ± 1.4 (i.e., between fairly light and somewhat hard).

### Strength and power performance changes

Table 3 shows strength and power performance changes after the RT program. The results showed that the RT program did not significantly improve the estimated full squat 1RM (*p* > 0.05; *g* = 0.40), and the percent change (9.2%) was below the MDC% (21.5%). As for the peak power, the RT program produced a small significant improvement (*p* < 0.05; *g* = 0.65), but the percent change (14.4%) was below the MDC% (22.4%). Regarding IHAS, the results showed that the RT program induced a small significant improvement (*p* < 0.001; *g* = 0.71), and the percent change (19.1%) was above the MDC% (14.6%). Finally, the results demonstrated that the RT program induced a small significant improvement in the CMJ (*p* < 0.05; *g* = 0.58), but the percent change (6.7%) was under the MDC% (7.2%).

**Table 3:** *Strength and power performance changes from pretest to posttest*.

	MDC (%)	Pretest (mean ±	Posttest (mean ±	*p*	Mean Diff (90%	%Δ (90% CI)	ES (*g*)
		SD)	SD)		CI)		
1(RM kg)	20.2 (21.5)	94.0 ± 18.7	101.7 ± 18.1	0.06	7.7 (1.9 to 13.5)	9.2 (2.5 to 15.9)	0.40
PP (W)	280.6 (22.4)	1251.5 ± 242.7	1414.8 ± 241.6	**0.01**	163.2 (74.7 to 251.8)	14.4 (6.3 to 22.5)	0.65
IHAS (kg)	6.3 (14.6)	43.0 ± 10.1	50.5 ± 10.0	**<0.001**	7.5 (5.3 to 9.8)	19.1 (11.9 to 26.3)	0.71
CMJ (cm)	2.7 (7.2)	36.8 ± 3.9	39.1 ± 3.9	**0.04**	2.3 (0.8 to 3.9)	6.7 (2.3 to 11.0)	0.58

MDC: minimal detectable change; %Δ: percent change; CI: confidence interval; ES (g): Hedges g effect size; 1RM: estimated one-repetition maximum in the full squat; PP: maximum peak power attained during the full squat test; IHAS: isometric hip adduction strength; CMJ: countermovement jump.

### Correlation between variables

**Figure 1:**
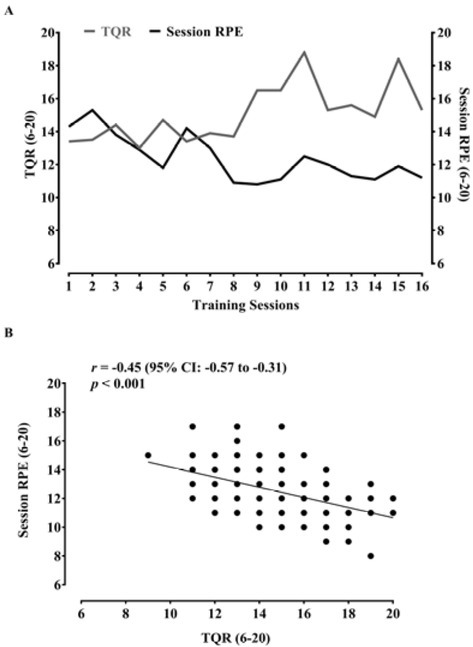
Total quality recovery (TQR) and the session rate of perceived exertion (RPE) over the 16 training sessions (A). Correlation between TQR and the session RPE (B).

Figure 1A depicts the pattern of TQR and the sRPE over the intervention, and Figure 1B shows a negative moderate significant correlation between TQR and the sRPE (*r* = -0.45; *p* < 0.001).

**Figure 2:**
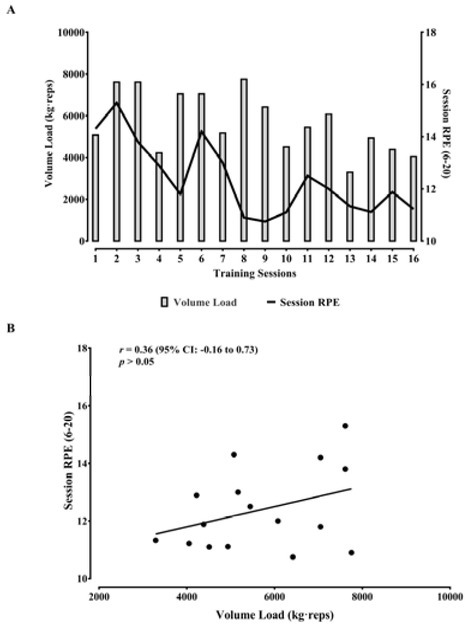
Volume load and the session rate of perceived exertion (RPE) over the 16 training sessions (A). Correlation between the volume load and the session RPE (B).

Figure 2A demonstrates the pattern of the volume load and the sRPE over the RT program, while Figure 2B shows that the sRPE and the volume load did not present a significant correlation (*r* = 0.36; *p* > 0.05).

**Figure 3:**
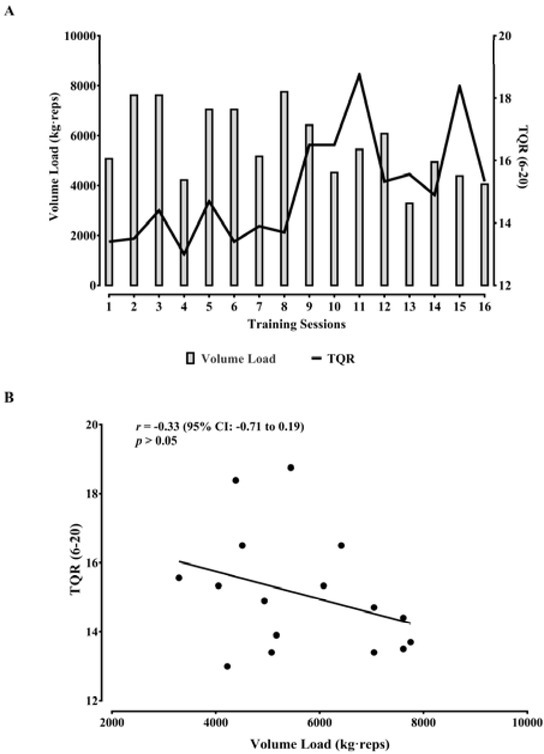
Volume load and total quality recovery (TQR) over the 16 training sessions (A). Correlation between the volume load and TQR (B).

Figure 3A exhibits the pattern of the volume load and TQR over the training sessions, and Figure 3B shows that TQR and the volume load did not present a significant correlation (*r* = -0.33; *p* > 0.05).

## Discussion

In this study, we aimed to examine (i) the strength and power changes during an in-season 8-week RT program in elite futsal players, and (ii) the associations between the sRPE with TQR and the sRPE and TQR with the volume load of the RT program. Although our results showed significant improvements in most physical performance outcomes, the gains did not surpass the MDC%, except for IHAS. Therefore, these results suggest that an in-season 8-week RT program based on low volume and low-to-moderate loads might not be a sufficient stimulus to induce meaningful changes in dynamic strength and power performance in elite futsal players. However, it was sufficient to improve isometric strength, partially corroborating our primary hypothesis. Furthermore, the secondary results indicated a significant correlation between the sRPE and TQR, but failed to demonstrate a significant association between the sRPE and TQR with the volume load, which partially confirms our secondary hypothesis. Therefore, monitoring TQR before sessions might be an effective strategy to predict elite futsal players' perceived level of effort in a RT session. However, the perceived recovery and effort measures do not present significant associations with the volume load of RT in elite futsal players.

A previous study with futsal players observed significant 1RM full squat gains after RT consisting only of the full squat (Δ = 17.0%) or combined with loaded change of direction exercises (Δ = 12.3%) (Torres-Torrelo et al., 2017). Although we employed a similar training design (i.e., low volume, low-to-moderate loads, and maximal velocities), 1RM gains were lower than those reported in the previous study and did not reach statistical significance, neither surpassed the MDC%. Similarly, although statistically significant, the full squat peak power and CMJ gains remained below the MDC%. Although the CMJ gains were similar to those presented by Torres-Torrelo et al. (2017) (Δ = 5-6%) and superior to those reported by Paz-Franco et al. (2017) (Δ = 0.9–1.5%), both with futsal players, the physical performance change was not sufficient to be considered a meaningful change for the development of the player's physical fitness. Possible reasons for the lack of meaningful changes might be associated with the RT program design or the player's physical condition at posttest.

Regarding the RT program design, due to the need to combine the RT with regular futsal training sessions without a significant accumulation of fatigue, the lack of loaded strength exercises or even insufficient volume or training loads might have attenuated strength and power gains. Although a RT program based on low volume and low loads using only the full squat might be sufficient to induce strength and power gains in futsal players competing in lower divisions (Torres-Torrelo et al., 2017), our results with elite futsal players do not suggest the same. Therefore, future studies should analyze whether prescribing more loaded strength exercises or increasing the training volume or load effectively improves strength and power in elite futsal players. On the other hand, it is essential to note that futsal players competing at the highest level face high workloads and great physiological demands throughout the season (Dogramaci et al., 2011; Makaje et al., 2012). Therefore, the posttest's dynamic strength and power gains might have been masked by accumulated muscle fatigue over the eight weeks. In this sense, possible alternatives to determine the actual performance would be to employ a 1–2 week tapering strategy and test players 2–4 weeks after the intervention (Morin et al., 2020) or measure the lifting velocity during RT sessions to estimate players’ daily dynamic strength and power performance (Pareja-Blanco et al., 2017). These strategies would be helpful to analyze the variations in strength and power over the intervention and examine each player's optimal adaptation period. Therefore, future studies might consider adopting either one or both approaches to effectively understand the time course of adaptations in dynamic strength and power during RT based on low volume and low-to-moderate loads in elite futsal players.

Interestingly, despite the non-significant increase in dynamic strength, there was a significant improvement in IHAS, and the gains were above the MDC%. Given that isometric actions are less frequent than dynamic actions, they are more sensitive to change when exposed to a RT stimulus (Carvalho et al., 2014). In addition, the implementation of the Copenhagen and full squat exercises for targeting the hip adductors might also have contributed to increasing IHAS. These results are relevant, considering that athletes with adductor strength deficits are at high risk of suffering from a groin injury (Moreno-Pérez et al., 2019). Therefore, strength and conditioning futsal coaches should consider targeting the hip adductors during RT to increase IHAS and eventually prevent groin injuries in elite futsal players.

Our study also analyzed the relationships between TQR and the sRPE and between the sRPE and TQR with the volume load. We found a significant negative correlation between TQR and the sRPE, suggesting that high TQR scores might result in low sRPE scores after a RT session. These results are in agreement with previous research that found a significant negative correlation (*r* = - 0.25) between TQR and the sRPE during a competitive period of nine weeks in national-level youth female basketball players (Cruz et al., 2018). Therefore, taken together, monitoring TQR and the sRPE might help coaches understand players' perceived recovery and how this state can influence their perceived level of effort during RT sessions. In addition, it can also be helpful to adjust the training prescription to get the most out of players.

Previous research demonstrated that higher volume loads resulted in a higher sRPE after RT sessions (Genner and Weston, 2014; Hiscock et al., 2018; Lodo et al., 2012). Although our results did not indicate a significant correlation between the sRPE and volume load, it was possible to observe that when the volume load was higher (i.e., week 1–4), sRPE scores were higher than those reported when the volume load was lower (i.e., week 5–8). Furthermore, this observation remains valid for the relationship between TQR with the volume load (i.e., higher volume loads, lower TQR scores, and vice-versa). Therefore, even though the correlations were not statistically significant, both relationships showed a particular pattern. Future studies with elite futsal players should extend the analysis to understand the relationship between the sRPE and TQR with the volume load during RT programs.

This study has several limitations. Firstly, the small sample size and the absence of a control group or an additional experimental group limit the results' generalization. However, it is essential to note that adding a control group within an elite team or selecting another experimental group with the same training routines and similar performance levels would be challenging due to ethical issues (Marques et al., 2008). Secondly, evaluating sprint, change of direction speed, and technical skills, such as kicking ball velocity, would be precious to measure the transfer effect of the RT program on physical and technical performance of elite futsal players. For example, performing a kinematic analysis during specific technical skills as observed in previous research (Gil et al., 2012) would be interesting to verify whether the RT program significantly improved these movement patterns. Finally, correlating training/match external load data acquired with GPS devices with strength and power measures would be essential to identify players’ strengths and weaknesses and adjust the workloads according to the player's needs. Therefore, researchers might consider the above limitations when planning future studies with elite futsal players to improve the evidence regarding the influence of RT on strength and power performance in elite futsal players.

## Conclusion

Performing an in-season 8-week RT program consisting of 2–3 sets x 3–6 reps at 45– 65% 1RM with maximal velocities in the full squat and complementary exercises with the same volume might not be a sufficient stimulus to induce meaningful dynamic strength and power gains in elite futsal players, but can improve isometric strength. In addition, monitoring TQR before every RT session might be a valuable and low-cost way to predict each player's perceived level of effort during RT sessions. This information may help strength and conditioning futsal coaches adjust training loads according to the player's needs.
